# On Your Own: Older Adults’ Food Choice and Dietary Habits

**DOI:** 10.3390/nu10040413

**Published:** 2018-03-27

**Authors:** Emily Whitelock, Hannah Ensaff

**Affiliations:** School of Food Science and Nutrition, University of Leeds, Leeds LS2 9JT, UK; emilywhitelock@hotmail.co.uk

**Keywords:** older adults, food choice, qualitative methodology, ageing, loneliness

## Abstract

The United Kingdom, in common with many countries, has an ageing demographic. Changes accompanying ageing can influence food choice and dietary habits. This study explored older adults’ perceptions and practices related to dietary behaviour and the factors influencing their food choice in later life. Semi-structured focus-group discussions were conducted with 30 individuals (aged 63–90 years) in a UK city. An inductive thematic approach was adopted for data analysis, and 4 themes and 12 sub-themes emerged: age-related changes (lower appetite, food changes, declining physical function); food access (food cost, support with food, maintaining independence); on your own (cooking for one, eating alone, shopping for one); and relationship with food (food variety, eating what you want, dieting). These influenced participants’ food acquisition, food preparation and cooking, as well as eating habits. Living alone and its substantial influence, as well as associated social isolation and loneliness, were highlighted in many of the discussions. Given the possible implications for nutritional intake, further work is recommended in this area. Likewise, steps should be taken to improve food access, increase opportunities for commensal eating and, fundamentally, address social isolation and loneliness in the older population.

## 1. Introduction

The demographics of the United Kingdom’s population are changing; between 2005 and 2015, there was an increase in over 65-year-olds by 21% and over 85-year-olds by 31% [1]. Globally, it is estimated that between 2015 and 2030 the number of over 60-year-olds will grow by 56% [2]. This change brings social and economic challenges, particularly concerning the healthcare of the growing older population [3]. Research gaps in ‘active and healthy ageing’ have been identified [4] and, specifically, there is a need to ascertain effective strategies relating to diet and nutrition for older people.

Nutrition is critical in its contribution to the health of older adults [5], and the likelihood of active and healthy ageing [6]. Physiological, psychosocial, and economic factors that can accompany ageing play a significant role in the nutrition and food choice of older adults, and can act as barriers to a healthy diet [7]. One of the main changes that can occur in later life is a reduced appetite. Common in older adults, this has been linked to changes to the digestive system, reduced saliva production due to medication, poor oral health, and reduced taste and smell acuity [8]. Slower gastric emptying [9], lower ghrelin levels [10], and possibly higher cholecystokinin and baseline leptin levels [11] all contribute to reduced appetite with earlier satiation. A poor appetite, often defined as the anorexia of ageing, can result in smaller portion sizes being eaten, changes in food choice, and weight loss [12].

Likewise, reduced taste and smell acuity can occur due to a normal ageing process [13], certain diseases, and medication use [14]. Reduced taste acuity can diminish food pleasure, and hence alter food choice and reduce appetite [14]. The impact of impaired olfactory acuity on nutritional status is unclear; previous research has reported no significant association [15], whilst one study found that older women with moderate to severe olfactory impairment have a significantly poorer diet quality compared to those with normal olfaction [16]. Taste, texture [17] and smell acuity losses in older adults potentially have an impact on dietary choice. When loss of appetite is combined with reduced taste and smell acuity, a lack of food cravings may result. Older adults can have fewer food cravings when put on a monotonous diet compared to younger individuals [18] and an absence of response to dietary monotony may contribute to an increased risk of malnutrition due to reduced food diversity. 

In addition, oral health and chewing efficiency tends to decline with age [19] and this can result in changes to food choice [20]. Reduced oral function due to tooth loss can lead to the avoidance of stringy, crunchy and dry solid foods [20], and in turn food avoidance can increase the risk of nutritional deficiencies. A systematic review of literature on older adults concluded that oral health alongside other risk factors may contribute to poorer nutritional health [21]. Furthermore, avoidance of certain foods due to chronic illness and associated dietary restrictions can result in poorer food choice [22], and it is estimated that 36% of 65- to 74-year-olds in the UK have a limiting longstanding illness [23]. Inherent in older individuals’ declining health is the increased use of daily medication, and polypharmacy is common [14]. Medication has been shown to reduce taste and smell acuity, lower saliva production [14], cause nausea, and reduce the desire to eat [24].

Declining physical abilities can compromise access to food, and mobility issues are thought to affect some 38% of people aged 70 and over in the UK [23] with about 6% of older people leaving their house once or less a week [25]. A recent study [26] identified store accessibility and service as affecting food choice and dietary behaviour for independently living Australians aged over 60. A New Zealand study [27] highlighted a lack of personal transport as a barrier to healthy eating for older men, which was nullified if reliable support networks were in place. In another study, data collected from nearly 400 older individuals in The Netherlands revealed convenience as a major influencer of food choice; travel time to the supermarket was shown to influence meal decisions significantly, whereas no significant effect of preparation time was found [28]. With many people now shopping for food online, mobility difficulties may be less of a barrier to food access in the future; however, at present approximately 3.8 million over 65-year-olds in the UK have never used the internet [23]. Additionally, sight loss is reported to affect 35% of people aged over 75 in the UK [23] and reduced vision can affect food access [29] through difficulties with activities such as food preparation, cooking and reading food labels.

One of the most substantial changes that can accompany ageing is bereavement and loss of a partner; this event has been related to poorer nutritional intake and diet quality [30,31]. Approximately 1/3 of over 65-year-olds live alone in the UK [23], and the last 20 years has seen a statistically significant 15% rise in older adults aged 65 to 74 years living alone; likewise, the number of people aged 75 and over living alone has increased by a significant 24% [32]. A recent study [33] found that diet quality in British men aged over 60 was greater in those who were married and not living alone. 

Food choice and dietary habits of older adults have been found to be affected by socioeconomic status, with lower scores associated with reduced diet quality [33], and socioeconomic measures during each life stage independently affecting diet in older age [33]. Previous work has also found the price of food to be a factor affecting food decisions made by older adults [26,27,28], and likely has greater weighting for individuals coming from areas of higher levels of deprivation. In the UK, 26% of older adults are ‘just about getting by’ with their income, with 27% of over 60-year-olds worrying about food costs [23]. It is thought that in the UK in recent years approximately 1 million individuals aged 65 and over have had to reduce the amount they spend on food shopping to be able to pay utility bills [23]; a trade-off in food versus fuel has been revealed in some older households during the coldest weather [34]. 

Against the backdrop of the UK’s ageing demographic and the acknowledged role of diet and nutrition in healthy ageing, this study’s aim was to explore the food choice and dietary habits of community-dwelling older adults within areas of high levels of deprivation. 

## 2. Materials and Methods

### 2.1. Recruitment and Participants

Recruitment was conducted in a northern UK city via community centres and day centres providing services for older adults, such as lunch clubs, breakfast clubs and social activities. Centres were identified according to the Index of Multiple Deprivation (IMD) for the areas that they served, and those in the lowest 2 deciles (most deprived 20%) were contacted. The centres provided access and supported researchers in recruiting participants to take part in the study. 

Focus-group discussions were selected as the main means of data collection. This was because they yield rich qualitative data, allow open discussion with the benefit of group dynamics, and can help to create lively discussion, aiding with recall. Focus-group interviews with 4–10 participants were considered optimal to encourage dynamic conversation and deter unmanageable discussion [35]. A challenge in conducting qualitative research is ensuring that the number of participants is small enough so that in-depth analysis can be completed and large enough to answer the research question [36] and include a range of perspectives [37]. To that end, recruitment and focus-group interviews were conducted until data saturation was felt to have been reached, and no further major points were arising in the discussions [37]. 

### 2.2. Focus-Group Interviews

A semi-structured format to the focus-group interviews was employed. An interview schedule was developed following a literature review, and particular emphasis was given to factors affecting food choice and changing food preferences from the perspectives of older adults. Questions were designed to be open, focused and not leading, in order to support data validity, reduce social desirability bias and encourage lively discussion. The interview schedule was reviewed by external academics and practitioners in public health nutrition and dietetics, local government, and a third-sector organization working with older adults. The interview schedule contained a series of questions and associated prompts covering food choice and diet, shopping and cooking, as well as eating out. An initial starter question asking participants about what they had eaten the previous day was used to introduce the topic and to make the participants feel at ease. To mitigate against crucial points being missed, each focus-group interview was concluded with participants being given the opportunity to introduce anything that they considered relevant and important that had not been mentioned. The interview schedule was refined during the study period to improve the delivery of the questions, aid comprehension, and support the quality of the data collected. 

In total, six focus-group interviews were conducted in June and July 2017. Each involved 4–8 participants except one focus-group interview which had 2 participants because of participant absences due to illness and appointments. Focus-group interviews were conducted by the researchers in a private room at the relevant community centre or day centre. Participants were provided with an information sheet outlining the details of the study and informed consent was obtained. Each focus-group interview lasted 45 min to 1 h, and entailed a brief overview of the process followed by an explanation of the ground rules, the semi-structured focus-group interview itself, and finally a closing statement. The discussions were led by the researchers using the interview schedule; questions, follow-on questions, and probes were flexible and dependent upon the progress of discussions and emergent issues. If there was ambiguity in answers, clarification was requested. Discussions were audio-recorded on two separate devices, and after each focus-group interview, the researchers discussed and recorded in writing any salient points.

### 2.3. Data Analysis

The focus-group discussions were transcribed using a denaturalised approach, which involved the correction of grammar, removal of interview noise such as stutters and pauses, and the exclusion of mannerisms that did not contribute to the content of the discussion [38]. Transcription of audio files inevitably introduces issues with accuracy and interpretation [39]; transcripts were checked carefully and InqScribe software (Inquirium LLC, Chicago, IL, USA) was used to support transcription. In addition, important thoughts were captured as researchers recorded any immediate observations. To reduce the influence of preconceived opinions and beliefs and to minimise potential effects on the data, reflexivity was practised by the researchers [36]. This included researchers’ acknowledgement of their impact, and scrutiny of their role in the study, e.g., identifying preconceived opinions and their possible influence, systematic discussion until consensus was reached, and memoing to capture reflections during data collection and analysis phases. 

Participants were assigned unique numbers in the transcripts, and when other individuals were mentioned by name by any of the participants, pseudonyms were assigned for anonymisation purposes. Transcripts were imported into NVivo 11 Plus software (NVivo 11 Plus, QSR International, Melbourne, Australia). This was followed by data analysis using an inductive approach, entailing data exploration, inductive coding and thematic analysis [40]. This involved listening to the audio-recordings multiple times, re-reading transcripts, and coding in an iterative fashion. An initial list of potential themes and sub-themes was created and then subsequently adjusted and refined during the analysis. Rigour in the analysis was supported through internal discussion and the review of codes by researchers, until the final set of themes and sub-themes described the data and captured the main emergent insights. Ethical approval (MEEC 16-016/16/2) for this study was granted through the faculty research ethics committee. 

## 3. Results

All participants were aged 63–90 years, with the majority being over 70, living alone, retired, White British and widowed (Table 1). Recruitment took place through centres located in areas considered the most deprived 20% in England; accordingly, most participants lived in areas of high levels of deprivation, with half of the sample coming from an area in decile 1 IMD (based on home postcode). Some participants, however, did not live in the areas in which the centers were located and the deciles for IMD for participants ranged from 1 to 7. 

**Figure 1:**
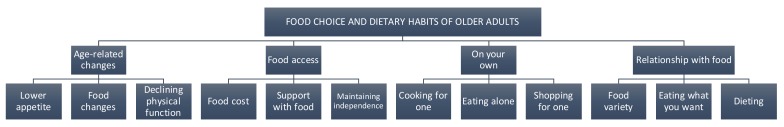
Older adults’ food choice and dietary habits.

Four themes and 12 sub-themes relating to food choice and dietary habits emerged from the data; these are presented in Figure 1 and described below. Representative quotations are also provided to substantiate the thematic findings; participants quoted are assigned unique identifiers (i.e., P1, P2, etc.).

### 3.1. Age-Related Changes

Age-related changes and associated impacts on food-based behaviours and decisions were reported by many participants. 

#### 3.1.1. Lower Appetite

Participants referred frequently to a smaller appetite, citing a reduction in the amount they ate, typified by smaller portions, with only a few reporting skipping meals.

*… I eat smaller meals, because I can’t eat … at one-time big plate like this [gestures piled-up plate]: lovely! But now it’s, you know, I just can’t face it anymore. (P24)* 

*I went for my dinner to my daughter’s on Sunday and I said, “Don’t pile my plate up because I won’t be able to eat it!” (P19)* 

*Yeah, you eat less. It’s like filling a car—you don’t put petrol [in] if you aren’t going anywhere. (P12)* 

*P8: So here [lunch club at community centre] we always say, “Smaller pile on my plate please” … but they don’t, and then we just leave it, but we don’t want to leave it. * 

*P7: It’s wasting, which is going against the grain for us; our age, we don’t waste nothing […] waste not, want not.* 

*P8: No matter what we say, they don’t change it [portion size]. * 

*P7: It out faces you …* 

Interestingly, participants discussed children’s portions and described how on a recent trip, some had selected children’s portions for their meal at a pub:

*We went to Bridlington on Friday, and we went into this pub for our dinner, and it were a child’s dinner. And it was really ample enough for an adult, and it were only 3 pounds something, and it were a child’s one. And all four of us had one and it were lovely. (P23)* 

#### 3.1.2. Food Changes

During discussions, participants reported how they avoided “heavy” or “acidic” foods due to digestion complications, or dry or chewy foods due to decreased oral health. Interestingly, participants also acknowledged changing food preferences, e.g., an increased liking for fish.

*When I was younger and [with] a family I probably ate more meat, but as I’ve got older I eat more fish and probably lighter meals, rather than heavy meals. (P27)* 

*Your taste buds change. Some food don’t taste the same as it used to. I think that’s why I dose it with salt. (P4)* 

*P7: I could do without meat. * 

*P9: I could do without meat too. * 

*P7: I like fish, but meat: I’m not bothered about … I think [it’s a recent change] … and I don’t know why … it’s probably the chewing! I don’t know. * 

#### 3.1.3. Declining Physical Function

For some older adults, declining physical function and reduced manual dexterity were reported to have a consequence on the effort put into cooking; this had implications on what participants cooked, often resulting in the preparation of simpler meals, diminished home baking, and a greater use of ready meals.

*I do have a lot of pain and I’d be sat there sometimes and be like, “I can’t be bothered, getting up and cooking a dinner now!” and I’d have a sandwich or something. (P8)* 

*I always used to sort of do wedding cakes and anniversary cakes, but as I’ve got older my hands won’t let me do that … So, I’ve lost interest in doing a lot of baking. I can’t do what I want to do, so I’ve just not bothered. I get so frustrated otherwise. (P24)* 

*I used to bake a lot, you know, apples pies, custard tarts and everything, and it all got eaten—but I don’t now for myself. I go to Morrisons (supermarket retailer) and look on the naughty counter … I think it’s [baking] too much hassle because I’ve moved into a flat and I’ve got an oven-cooker, whereas in my house my cooker was there [standing height], but now it’s down there [near the floor] … and it’s a problem getting the stuff out, sat down getting it out. (P7)* 

### 3.2. Food Access

It was clear that food access was relevant to participants; this encompassed several aspects including the cost of food, support with food shopping and meals, e.g., transport to supermarkets, meals on wheels, as well as trying to maintain independence when it came to food acquisition. 

#### 3.2.1. Food Cost

Many participants intimated that food affordability was an issue, referring to the cost of food, cost of transport to do food shopping, and the cost of eating out.

*I always used to go for a brand name, a particular one, but I keep trying now the cheaper one [supermarket’s own brand]. (P16) * 

*Well when I go into town I eat at McDonald’s or I go to the pound bakery and buy stuff there, because it’s so expensive to go and eat in a café now. Years ago when I was working I’d go to a café and eat, but not now. (P10)* 

*Well I go to Aldi and I usually try to go early in the morning because of the reduced stuff. You can get meat 30% off or 50% off … if you are lucky you find. (P12)* 

*I usually go to Tesco or Aldi; I prefer Tesco—it’s the first place I go. I have my list and my wheelie trolley. When I go to Tesco, the first place I go is the reduced counter. Have a look there, and if there’s anything worth buying I’ll plan my meals around that. And I go to Fultons (value food store) as well because they do some good bargains in there. (P10)* 

#### 3.2.2. Support with Food

Almost all participants received some support with food, typically provided by family, friends, or community services. Generally, food shopping was carried out by participants, with transport to the supermarket or shops provided by others.

*I used to go [food shopping] with a friend that I had, and she drove a car and I don’t. We used to go somewhere out for the day and then go to the supermarket. But unfortunately, she died recently. So, my son started getting it [food shopping] for me. (P8)* 

*I shop once a week at Morrisons on the Access Bus (door-to-door bus service). It picks us up and drops us off. We have an hour shopping … and I do my weekly shop there. (P7)* 

Individuals reported varying levels of support with meals; some ate all main meals at lunch clubs, others had one or two meals a week prepared by family members, and a few prepared all their meals independently. Takeaways did not feature in typical food acquisition and, generally, eating out occurred as part of trips organised by community centres, or occasionally with family and friends. Carveries in particular were highlighted, and this participant explained how they offered a meal that was seldom on the menu at home if you lived on your own:

*The beauty of going to a carvery when you’re on your own is that you have a choice of a roast joint, and I think everyone would possibly agree with me on this one: we buy chops, or mince, or we buy pieces of steak because you can’t buy a joint for one …. because if you buy a small joint and roast it, it ends up looking like an Oxo cube—it shrinks. If you go to a carvery you’ve got the benefit of this lovely roast which is what we did years ago as a family. (P17)* 

#### 3.2.3. Maintaining Independence

Despite most participants receiving some element of support with food (generally food shopping or transport to the shops), maintaining independence and a degree of autonomy over food decisions was important.

*You don’t want someone arranging your life for you. You want to decide for yourself. (P1)* 

*It’s about maintaining your independence. (P4)* 

### 3.3. On Your Own

In all focus-group interviews, participants talked about being on their own, and it was clear that this had a substantial impact on their eating, shopping and cooking habits. 

#### 3.3.1. Cooking for One

Being on your own and cooking for one resulted in the preparation of simpler meals (including ready meals), with participants often referring to being “fed up of cooking for myself” and not being “bothered”, as it was just for them—unlike when “it was for the family”.

*Unfortunately, I woke up one morning a widow so I thought I might as well use the microwave. I just couldn’t be bothered. (P30)* 

*When you’re on your own you just can’t be bothered, you know. (P28)* 

*P19: I do think you neglect yourself when you’re on your own. Definitely, yeah. * 

*P18: Because you know you’ve got to make it if someone’s there, don’t you. * 

*I think I could just about still make a roast dinner if I had the need to, but I haven’t done it for years because you don’t cook like that when you’re on your own. (P17)* 

*P27: It’s motivation I think really: “Owh, I’m on my own—it don’t matter.”* 

*P28: You get like that … * 

*P27: But I think sort of spending a bit of time cooking … it can be lonely, you see, when people live on their own.* 

*P28: Yes it can.* 

*P27: Especially at meal times you know.* 

#### 3.3.2. Eating Alone

Closely related to the previous sub-theme, eating alone was perceived negatively and something that led to a less enjoyable meal time, compared to eating with others.

*We go out on a Wednesday, bowling or something like that, then we usually enjoy a meal. You go through it [the food] but you don’t realise the time’s gone, whereas you sit on your own [pretends to stare into the distance] … (P20)* 

Interestingly, several participants discussed how they would cook a meal and then not feel like eating it.

*If I’ve done myself what I call a ‘proper big dinner’, I’ll cook it and put it on my plate and look at it and think, “I don’t really want that now”. (P24)* 

When this was explored further in the discussions, it became apparent that this was attributable to eating alone.

*P20: … I have my grandkids round at the weekend and that’s much better because I can sit with them. And I’ve cooked them a meal and probably on a Sunday or something we’ve had a big roast dinner […] You enjoy it because someone else is there, but sat on your own sometimes it’s …* 

*P24: It [food] looks a bit overpowering sometimes, don’t it?* 

*P14: My wife used to do it [cooking]. I can cook but I just can’t be bothered. When you do it for yourself, by the time you’ve done it you don’t feel like eating it … well, I don’t. * 

*P16: That’s true actually. * 

*P17: You don’t enjoy it the same when you’re sitting and eating on your own because there is no one to say sort of, “That was nice” or “I’ll do the dishes”. It’s the other bits that go with it isn’t it, the social part of it. * 

Participants were clear about the negative aspects of being on their own, and this was particularly the case when talking about eating alone; although not explicitly referred to, the issue of loneliness emerged during many discussions.

*P20: … although he [husband] was very awkward with his food there was still somebody there. We didn’t always have the same meals but you’ve got somebody there. Somebody else in the room with you, and you were eating. * 

*P21: Yeah, it makes a difference […] You’ve sort of lost the incentive. * 

*P20: I sometimes think when you’re on your own and you sit down the moment’s gone. * 

*P21: Mind you that’s the same … when they were sat in the chair and you were maybe sat reading or knitting but they were there in the chair … and it’s that sort of feeling. * 

*P20: Yeah that’s it … and even if there was no sort of communication there was somebody there. * 

*P21: And [now] you end up talking to yourself. * 

*P20: There was still somebody there, but now you’re sat there.* 

*P21: It’s amazing what difference it makes i’nt it? * 

#### 3.3.3. Shopping for One

Participants discussed adjusting to shopping for one having previously bought food for the whole family. Most participants bought most of their food shopping at supermarkets, and one of the main issues was packet sizes being too large. This would result in food wastage—which was something to be avoided as they “were brought up not to waste”; and so this meant that certain foods were less likely to be purchased. Vegetables were the food group that participants had the greatest difficulty with when buying for one.

*Sometimes when you’ve cooked for family throughout your life and then all of a sudden you’re on your own, you’re sat on your own, you’ve got to sort of think, “I’m just doing it for me”. I found that hard, to stop shopping for a family when there were only me. (P26)* 

*I find when you buy packs of vegetables from Morrisons there’s too much in them. I love spring cabbage and I haven’t bought any for ages because there’s a load of it. (P7)* 

*P16: There’s not much in supermarkets for one. * 

*P14: A lot of things are in packs. * 

*P17: You can buy an orange, apple, grapefruit and snap a banana off—but the rest of it’s in packs. * 

*P15: Even parsnips, things like that; you don’t want a pack of 6 or 7 parsnips. I just want one. * 

*I like small portions because if I get a big bag of potatoes they end up sprouting. (P1)* 

*We’ve found Morrisons is a little bit expensive. “Buy 2 for 1”; we don’t want two. There are a lot of things like that in Morrisons. (P12)* 

Whilst some participants went to the supermarket once a week to do their main food shop, a number of participants explained that they went multiple times a week or every day; this was explained as something to do and a reason to leave the house.

*That’s the biggest problem when you’re on your own—boredom. It’s no good sat there watching the television all the time. Watching the television all day, I call it a ‘no day’. You’re just sat there watching the telly like a zombie. (P12)* 

*Well I go [food shopping] every day. Mostly it’s something to do, you know. I know it’s a bit sad. (P14)* 

*I try and go shopping every day, basically because it gets me out of the house. (P10)* 

### 3.4. Relationship with Food

Each participant’s relationship with food was individual and based on previous experiences and food history. However, commonalities did emerge and these are outlined below.

#### 3.4.1. Food Variety

Buying novel produce was uncommon and participants rarely strayed from their habitual food purchases, and frequently mentioned sticking to what they know and enjoy. On several occasions, participants raised the importance of eating a varied diet, which was referred to in terms of not eating the same things every day. When it came to trying new foods, some participants expressed an unwillingness, while a few were willing but only if someone else cooked it.

*I like interesting meals and I like varied meals. I don’t like the same thing over and over again. (P21)* 

*I tried scallops the other day. They’re quite nice aren’t they! I wouldn’t cook it though—I went to someone’s Christmas party. (P1)* 

Interestingly, some participants referred to feeling “bored” with their food or looking in supermarkets for “something different”:

*P18: You could get a tin out and make a sandwich, but now I look and I think, “I don’t want that” […] I think it’s because you’re bored, I don’t really know. * 

*P19: I think it’s boredom … because you, I don’t know, you just seem to eat the same sort of food day in day out, sort of thing. * 

*I like interesting food. I like interesting meals and I like varied meals. I don’t like the same thing over and over again. I’d eat it, don’t get me wrong, I’d eat it, but I would wish for a change. (P21)* 

#### 3.4.2. Eating What You Want

Many participants expressed choosing and eating foods based on enjoyment, and food choices appeared to be mostly hedonic rather than for any other reason. There was also a general sense that this was different to before, with some participants portraying an attitude of being able to now choose food as they please.

*I’ve got a very sweet tooth, I’ve not got a savoury tooth. Well I eat things I shouldn’t eat really; but if I want it I have it. (P4)* 

*… I used to look at calories, once upon a time, but not now. Not watching my figure now! (P1)* 

*I think you come to a stage you think, “Right that’s it, enjoy yourself!” … But as I say: in moderation. I like most things, especially if they’re cooked for me. I’d eat anything … I eat more or less anything. (P14)* 

*… They all say: a little bit of what you fancy does you good. (P16)* 

#### 3.4.3. Dieting

Interestingly, across all focus-group interviews and to varying degrees, participants talked about their weight and dieting, with described efforts focusing on avoiding foods considered high in fat and high in sugar.

*I think once you get to 60 you find it really hard to get your weight off, don’t you? (P18)* 

*I’ll go as far as saying, I found it much, much easier to stop smoking than diet. And I just said, “Right that’s it; I’m having no more!” and no more did I have. Now I couldn’t do that with a diet! (P19)* 

*I put weight on very quickly, I always have done—so I now make a conscious effort. I’ve yo-yoed, my weight all my life. But I think I’m realising now you can still eat and eat well without the rubbishy food. But I still have it, everything in moderation. A little bit of what you fancy does you good. So I try to keep it that way. (P15) * 

For some, trying to lose weight was considered an ongoing issue; for this participant it was for a specific reason:

*I did it [went on a diet and lost weight] when they told me I needed a new hip, didn’t I. I did it then. They didn’t do my hips, so that disheartened me. So now I think I will do it because I’m due back to see them in December. (P18) * 

## 4. Discussion

Several factors influenced older adults’ food choice and dietary behaviour. Together, the themes described, their component sub-themes, and their interrelationships affected food acquisition, food choice, food preparation and consumption. Differing levels of prominence were seen for different aspects of food-related behaviour. In particular, being on your own and the unspoken associated loneliness affected participants’ food practices at home, while food access focused on the acquisition of food from supermarkets. 

In this study, being on your own influenced participants’ eating occasions and was seen to contribute to diminished effort with meals. This was generally attributed to “being on your own”, not having anyone else to cook for and “not being bothered”. This lack of motivation to cook has been reported previously in older women who had lost their partner [41], and is pertinent given that motivation is an influencing factor in healthy eating, when cooking for one and eating alone [42]. Many participants also reported cooking less frequently and cooking simpler meals in later life compared to previously. This transition in food choice to incorporate simpler meals that are easy to prepare (including ready meals) is notable, given the potential implications on dietary intake and nutritional risk. The change observed in this study reflects a previous study conducted in Sweden, which found that for some older women now on their own, the importance of providing a complex meal diminished, and preparing and cooking a meal, previously considered similar to a gift, lost its meaning [43]. 

As well as preparing simpler meals, many participants in this study reported a lower appetite and consuming less food in later life—with smaller meals the main associated change to their food practices. Interestingly, many referred to having to request smaller portions when eating away from home, e.g., at family or community centres. The issue of children’s portions on menus was notable, with a group of participants having made use of this during a recent trip. Between the ages of 40–70 years food intake reduces by approximately 25% [9] and declining appetite is considered to develop as a result of a reduced drive to eat and differences in satiety signaling [12]. Older adults’ smaller appetites and portion sizes being too large were reported by participants in a previous qualitative study [41]. Although unusual in the UK, some restaurants in the US cater for older adults specifically with a ‘senior menu’; this contains smaller portions, often with ‘healthier’ sides, and costs less than similar options from the main menu. 

All participants in this study were recruited through community centres or day centres providing support to older adults, and each participant had their own individual level of community, social and familial involvement. Despite their connections with the centres, some participants talked about being on their own and their limited familial contact. They also described efforts to improve social contact (e.g., shopping every day), indicating a feeling of a certain level of social isolation. The relationship between food habits, reduced commensal eating and being on your own was highlighted in this study. Living alone has been reported to contribute to social isolation, with 12% of over 65-year-olds feeling cut off from society [23]. This is specifically relevant to dietary habits as both living alone and social isolation can result in reduced commensal eating and, in turn, a lack of cues to eat. Previous research has also found living alone to be associated with higher nutrition risk in Australian older adults [44]; another study found greater social isolation to be associated with increased nutritional risk in American older adults [45]. Recently, social engagement has been highlighted as a potential lever for change, alongside psychological factors, in promoting better diet in older adults [41].

This study’s findings indicate that living alone impacted on participants’ eating occasions. In particular, several participants discussed how they might prepare a meal for themselves, to then not feel like eating it and the meal being “overpowering”. Participants attributed this to eating alone, and distinguished it from eating commensally. This is an interesting finding and adds to existing literature; it is proposed that these incidences bring home the reality of being alone. A recent Canadian study suggested eating alone may act as reminder of bereavement and result in reduced pleasure from eating [46]. These incidences may also be indicative of participants feeling a sense of lacking or missing social and/or familial involvement; although not explicitly referred to by participants, loneliness emerged in many of the focus-group discussions. This is pertinent, given that nearly 1 million over 65-year-olds in the UK often or always experience loneliness [23]. Furthermore, research points to the impact of loneliness on nutritional status [47] and identifies loneliness and bereavement as contributing to food choices and nutritional intake in older adults [48]. In addition, social isolation and loneliness correlate with the development of depression [49], and nutritional deficit and depression are associated in older adults [50].

The level of social support varied substantially between participants in this study, and this had an influence on access to food. Many participants relied on Access Buses to get to supermarkets, thereby dictating their shopping location. The relationship between increased support with food and reduced individual food autonomy and, consequently, an impact on dietary habits is interesting to note. Likewise, the desire to maintain independence was highlighted by some participants in this study, with many expressing the importance of autonomy in their food decisions. Participants’ desire to maintain levels of independence corresponds with another study of older adults in Australia which identified a strong determination to maintain independence, including around shopping and meal preparation [26]. However, while the Australian study reported the occasional use of convenience-type foods, this was not the case in this study—possibly reflecting differences based on cultural and/or socioeconomic status between the two study samples. 

In this study, the cost of food emerged as a key consideration in participants’ food choice. This was relevant in food acquisition as well as food activities e.g., selecting where to eat outside the home. This was to be expected based on the areas of high levels of deprivation from which the sample was recruited. The relevance of food cost found in this study reflects previous research where the price of food was found to be a factor affecting food decisions made by older adults [26,27,28]. This finding also reflects current concerns relating to food cost within the older population in the UK [23]. 

Participants reported changes in their diet that revolved mainly around reduced meat consumption, a growing preference for fish, and the avoidance of heavy foods. These changes were attributed to a deterioration in oral health and avoiding foods that were hard to digest or needed chewing. The impact of such changes on nutritional intake is not known and worthy of further work. This is particularly so given that for older men from a low-income population, similar changes have been found to be associated with reduced diet quality [51]. Elements of these changes, specifically the changing preferences for meat and fish, were found in a study that explored the changing dietary choice for older Irish adults [52]. 

In this study, participants also relayed a sense of choosing and eating foods based on enjoyment rather than anything else, and this was qualified as being different to before and almost an earned entitlement now. Consequently, food choices were based on hedonic attributes and many expressed a preference for energy-dense foods, particularly sweet bakery products. Eating what you wanted dominated considerations and, for example, health aspects were not a key factor in food-based decisions. This emphasis on taste and sensory appeal has been found previously [28,53]; its predominance to the exclusion of other considerations contrasts with previous work and adds to the existing literature. Eating to improve quality of life has been reported to be a motivator for older Irish adults to consume a healthier diet [52], and likewise a study in The Netherlands looking at major motivators of food choice in older adults from both high and low socioeconomic groups, identified healthiness as the most important attribute for all participants [28]. This difference may be due to cultural differences and/or differing levels of socioeconomic status, as the Dutch research identified healthiness to be more relevant in the food decisions made by those of a higher socioeconomic status [28]. 

Reduced physical function and manual dexterity were linked to participants’ preference for simpler meals that were easy to prepare. This preference for simpler and more convenient foods that were easy and quick to prepare was related to more than one factor in this study and, for example, was also related to cooking for one. It also indicates a transition in food practice and, likewise, has possible implications on dietary intake and nutritional status. The importance of food convenience as seen in this study has been shown to be a key factor in home-bound older individuals’ food choice [53]. 

Changes reported by participants related to how food was chosen, prepared and consumed. Of particular relevance is the significance of the change in social aspects, e.g., reduced commensal eating, which was seen to influence foods consumed, and appeared to be driving food-related behaviour, e.g., daily food shopping. Participants’ food shopping every day explained as “something to do” is an interesting finding, and may reflect the changing role of food shopping for older adults, i.e., with a stronger social purpose, and may be indicative of social isolation and, more importantly, participants’ loneliness. These changing needs and their impact on subsequent food practices are interesting and worthy of further examination. Furthermore, this may be a key factor in determining implications on practice and policy. A study examining women’s experiences of cooking and food preparation in later life reported that a reduction in cooking may be due to priorities changing, and the value placed upon social engagement [54]. 

Participants in this study described still feeling inclined to have a traditional roast dinner once a week and, linked to this, they expressed their enthusiasm for carveries which were the most visited place for eating out. This may be related to associations between a “traditional roast dinner” and an enjoyable family social occasion and, interestingly, a number of participants still cooked elaborate roast dinners when their family came to visit, with past experiences with food influencing participants’ current food choice behaviour. Previous food experiences have a large effect on current food choice by older adults [41,52] and food choice trajectories may withstand transitions due to the momentum from repeated food choices and habitual food selections [55]. 

As well as advocating eating what they wanted, participants in this study referred to being overweight and needing to lose weight. This is in contrast with growing concerns over malnutrition and unintentional weight loss in older adults in the UK [56] and also contrasts with previous work [57] which found that those from areas with a lower IMD were at an increased risk of malnutrition. The relationship between participants’ selection of foods primarily based on enjoyment and their weight status is unclear, and would be interesting to examine further. Similarly, an exploration of food choice in older adults from areas of lower levels of deprivation would be useful in examining whether this emphasis on losing weight remained. 

Overall, changes in food-related activities for participants in this study reflect modifications in individuals’ food choice trajectories, either as a transition or turning point. Similar influences of widowhood and the development of chronic illness have been reported previously [55], as have the impacts of retirement, bereavement and medical conditions on diet [41]. A household’s diet negotiations profoundly change following disruption of the household due to death [58], and food behaviour following widowhood in later life has been described as a two-stage process entailing the re-establishement of personal food systems [59]. The relationship between food habits (in particular reduced commensal eating), social isolation and loneliness highlighted in this study is worthy of further research. In particular, the subsequent impact on older adults’ food choice and nutritional status should be examined. 

It is interesting to note that participants in this study were recruited through centres providing support to older adults, thereby most likely excluding those experiencing the highest levels of social isolation. Further research with individuals not accessing community support would be valuable, as would a focus on older males living alone who are more likely to have a less extensive social network and potentially be involved in fewer social activities compared to their female counterparts [60]. This is particularly relevant, given that greater engagement in social and cognitive leisure activities by older adults has been associated with having a better quality diet [61].

The limitations of this study should be acknowledged. The diversity of viewpoints expressed by participants may have been limited due to the study’s geographical focus, i.e., one city, and the centres’ serving areas (lowest 2 deciles for IMD), and may not represent the diversity of the older adult population group, which is acknowledged as a highly heterogeneous group. Sample bias due to recruitment being limited to older adults frequenting community and day centres is another limitation. As recruitment was from within centres, individuals within focus-group interviews knew each other and this may have affected the accuracy of responses [35]. Social desirability bias and its influence on responses should also be acknowledged. 

### Implications for Policy and Practice

To support healthy ageing and inform respective policy and practice, it is essential that older individuals’ needs are addressed better and identified barriers are reduced. One of the barriers identified in this study related to large packet sizes in food retailers. This, combined with older adults’ avoidance of food wastage, resulted in their avoidance of certain foods (notably vegetables). A need for smaller packet sizes at the same value for money as larger ‘family packs’, should be considered by food retailers and producers. Similarly, smaller portion dishes within the food service industry should be routinely available. This would respond to the demand as seen in participants’ use of children’s portions on menus, and would correspond to ‘senior menus’ seen elsewhere. Smaller portion dishes costing less than similar options from the main menu could meet reduced appetite as well as food cost considerations. Furthermore, it is recommended that smaller portion dishes are developed with appropriate consideration to nutrient density (rather than simply decreasing portion size). Both smaller portion dishes in the food-service industry and smaller packet sizes in food retailers have the potential benefits of reducing food waste, improving diet variety and food choice for consumers, whilst also enabling the food industry to respond to changing customer needs.

Participants reported the importance of ‘eating what you want’ and choosing food based on taste and sensory appeal. This emphasis could be used as a focus in targeted strategies relating to better food choice and dietary habits in older adults, e.g., publicity for lunch clubs, promotion of food preparation, and cooking at home. 

During many discussions, the issue of being on your own was highlighted by participants. The influence of this on participants’ food acquisition, preparation and cooking as well as consumption were evident. The pervading influence of being on your own is considered an important finding of this study, and adds to the growing literature related to older adults’ food choice and dietary behaviour. Furthermore, the issue of loneliness emerged in particular when discussing meal preparation. This reflects ongoing concern surrounding loneliness in the older population in the UK [62,63]. Strategies to address the levels of loneliness experienced by some older people and its substantial influence on their day-to-day lives should be pursued. There is a need to raise levels of awareness with the wider population, including family, friends and carers. Likewise, the expansion of and introduction of new befriending schemes (such as those developed by Age UK) is required. In addition, an expansion of lunch clubs in community and day centres is recommended. Such activities promote commensality and social engagement for those attending; an exploration of the accessibility of such services and potential barriers should also be considered.

## 5. Conclusions

This study has provided valuable insights into everyday food practices in later life, and in particular has emphasised the substantial influence of living alone. It has highlighted age-related changes, food access, relationship with food, and being on your own—and how these factors play a role in older adults’ food acquisition, preparation and consumption. Participants’ accounts of being on their own, their experiences of this, and the influence of this on their daily food habits is a key finding. Related to this, social isolation and loneliness emerged in many of the focus-group discussions and reflects ongoing concerns surrounding these issues and the imperative to act. Given the critical contribution of nutrition to healthy aging, strategies to promote better dietary behaviour are needed. Furthermore, initiatives need to address direct issues of food access as well as contributory elements such as loneliness and social isolation within the older population in the UK. 

## Figures and Tables

**Table 1:** Demographic characteristics of the participants in the focus-group interviews.

Characteristic	*N*
Gender	
Male	5
Female	25
Age (years)	
60–65	1
66–70	7
71–75	4
76–80	10
81–85	6
86–90	2
Ethnic origin	
White British	29
White Irish	1
Employment status	
Retired	28
Unemployed	2
Relationship status	
Married	3
Divorced	5
Separated	1
Single	3
Widowed	17
In a relationship but not cohabiting	1
IMD decile *	
1	15
2	4
3	3
5	2
6	3
7	1
Number of people in household including self	
1 Adult	26
2 Adults	2
3 Adults	2

* IMD: Index of Multiple Deprivation, Decile 1 is the most deprived 10%; decile based on home postcode; 2 participants are not included due to misgiven postcodes.
